# A Novel Lightweight Authentication Scheme for RFID-Based Healthcare Systems

**DOI:** 10.3390/s20174846

**Published:** 2020-08-27

**Authors:** Feng Zhu, Peng Li, He Xu, Ruchuan Wang

**Affiliations:** 1 School of Computer Science, Nanjing University of Posts and Telecommunications, Nanjing 210023, China; zhufeng@njupt.edu.cn (F.Z.); xuhe@njupt.edu.cn (H.X.); wangrc@njupt.edu.cn (R.W.); 2 Jiangsu High Technology Research Key Laboratory for Wireless Sensor Networks, Nanjing 210003, China

**Keywords:** lightweight, authentication, radio frequency identification, healthcare systems, security

## Abstract

The Internet of Things (IoT) has been integrated into legacy healthcare systems for the purpose of improving healthcare processes. As one of the key technologies of IoT, radio frequency identification (RFID) technology has been applied to offer services like patient monitoring, drug administration, and medical asset tracking. However, people have concerns about the security and privacy of RFID-based healthcare systems, which require a proper solution. To solve the problem, recently in 2019, Fan et al. proposed a lightweight RFID authentication scheme in the IEEE Network. They claimed that their scheme can resist various attacks in RFID systems with low implementation cost, and thus is suitable for RFID-based healthcare systems. In this article, our contributions mainly consist of two parts. First, we analyze the security of Fan et al.’s scheme and find out its security vulnerabilities. Second, we propose a novel lightweight authentication scheme to overcome these security weaknesses. The security analysis shows that our scheme can satisfy the necessary security requirements. Besides, the performance evaluation demonstrates that our scheme is of low cost. Thus, our scheme is well-suited for practical RFID-based healthcare systems.

## 1. Introduction

The Internet of Things (IoT), as its name implies, means to connect a large number of objects to the Internet, such as smartphones, vehicles, sensors, and wearable devices [1]. Nowadays, IoT has gradually penetrated into our daily life, providing services and resources in various domains, including healthcare, smart cities, home automation, smart grid, industrial manufacturing, logistics, business management, and intelligent transportation [2,3].

One of the fundamental technologies of IoT is radio frequency identification (RFID) [4]. RFID uses radio waves for short-range communication so as to provide contactless and automatic object identification [5]. A typical RFID system consists of three components: RFID tag, reader, and server. In the system, each tag is attached to an object and usually stores the information about the object. The reader plays a role as the intermediary between the tag and the server. To identify an object, the reader first retrieves the object information from the tag and then sends it to the server for further processing.

**Figure 1:**
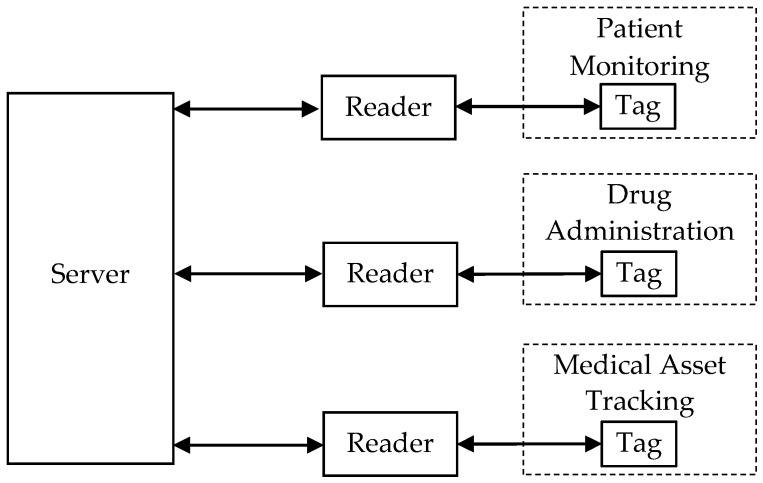
A typical radio frequency identification (RFID)-based healthcare system.

With the nice feature of noncontact automatic identification, in recent years, RFID technology has been applied in healthcare systems for providing intelligent services such as patient monitoring, drug administration, and medical asset tracking [6]. The architecture of a common RFID-based healthcare system is demonstrated in Figure 1. A patient in the system is given a wearable device (e.g., a smart wristband) that contains a sensor and a tag. The sensor in the wearable device collects the patient’s medical data and then stores it in the tag. A nurse can read the patient data from the tag using a reader. The data is then transmitted to the server so that doctors can remotely access the patient information, which helps with the goal of real-time patient monitoring. In addition, medication errors [7] caused by inadequate patient monitoring can be reduced. Drugs are also attached with tags so that medical staff can easily check their integrity and availability with a reader. Medical staff can further verify whether the right drug is being given to the right patient. According to the U.S. Food and Drug Administration [8], the improvement of drug management can also help reduce the number of medication errors. By integrating with RFID technology, hospitals can track medical assets in order to mitigate theft loss, improve resource utilization, and save costs [6]. Thus, patients and medical staff can benefit a lot from these services.

Although an RFID-based healthcare system has lots of advantages over a traditional one, it suffers from new security and privacy risks [9]. For example, if an adversary can track a tag embedded in the smart wristband of a patient, the location of the patient is known by the adversary. Furthermore, an adversary may impersonate as a legitimate reader to collect a patient’s medical data from the patient’s smart wristband, leading to medical privacy leakage. Hence, a suitable solution to secure RFID-based healthcare systems is urgently needed.

RFID systems have two common architectures. One is that the reader is fixed and has a wired connection to the server. The other is that the reader is portable and connects to the server wirelessly. In the former one, there is a special cable for the connection between the server and the reader so the channel is considered to be safe, while the channel in the latter one is deemed to be insecure due to the wireless connection between the server and the reader [10]. With the advances of mobile technology, the second architecture has become the mainstream of RFID systems so our article mainly considers this architecture. Besides, in either architecture, since the reader and tag use radio waves for communication, the channel between them is unsafe.

A straightforward idea for securing an RFID system is to encrypt all the communications. However, in practical RFID systems, especially the large ones, tags conforming to the Electronics Product Code Class-1 Generation-2 (short for EPC C1G2) standard [11] are most widely used due to the low price. EPC C1G2 tags have limited computation power and storage capacity and thus only support restricted operations such as exclusive-OR, cyclic redundancy check calculation, and pseudorandom number generation. Besides, such low-cost tags usually have no more than 2000 equivalent gates available for security purposes [12], which is insufficient for standard cryptographic algorithms. For instance, the smallest known implementation of the Advanced Encryption Standard (AES) algorithm needs 2400 equivalent gates [13]. Therefore, a lightweight security solution is required to secure RFID-based healthcare systems.

To address this requirement, in 2019, Fan et al. [14] proposed a lightweight RFID authentication scheme in IEEE Network. They stated that their scheme can provide strong security for low-cost RFID-based healthcare systems. In this article, we first show that their scheme has several security flaws and then propose our improved scheme.

### 1.1. Contributions

We make the following contributions to this article.

- We perform a security analysis of Fan et al.’s scheme [14] and demonstrate that this scheme fails to support forward secrecy and is prone to impersonation attacks.
- To overcome the security vulnerabilities of Fan et al.’s scheme, we propose an improved scheme. The security of our proposed scheme is evaluated from informal and formal security analyses. The analysis results illustrate that our scheme can offer better security than existing schemes.
- To show the efficiency of our proposed scheme, we compare it with other existing schemes in terms of computation cost, communication cost, storage cost, and hardware implementation cost. The performance evaluation results present that our proposed scheme is lightweight and conforming to the EPC C1G2 standard.

### 1.2. Organization

The rest of this article is structured as follows. Section 2 briefly discusses the related works. Section 3 presents the preliminaries, including the security demands, adversary model, and notations used in this article. Section 4 firstly describes Fan et al.’s scheme [14] and then analyzes the security of this scheme. Section 5 proposes our enhanced scheme, followed by its security analysis. Section 6 evaluates the performance of our proposed scheme. Finally, Section 7 summarizes the paper.

## 2. Related Works

Over the last several years, researchers have proposed a variety of authentication schemes, aiming to secure RFID-based healthcare systems. In 2014, Zhao [15] proposed an RFID authentication protocol based on elliptic curve cryptosystem (ECC) to secure communications in healthcare environments. In the same year, Zhang and Qi [16] proposed an ECC-based RFID authentication protocol for medical systems to enhance patient safety. However, Farash et al. [17] analyzed the protocols in [15,16] and pointed out that these two protocols cannot ensure forward secrecy. Farash et al. also suggested an improved protocol based on ECC to enhance the security of healthcare environments in [17]. Later, researchers proposed more ECC-based RFID authentication protocols [18,19,20,21,22,23] for healthcare applications. Because of the high hardware requirement of ECC, these ECC-based protocols are not well compatible with the EPC C1G2 standard.

In 2015, Srivastava et al. [24] proposed a new authentication protocol to strengthen the security of telecare medicine information systems (TIMSs), which is based on a hash function and shared secrets. However, Li et al. [25] analyzed Srivastava et al.’s protocol and found that an adversary can use a stolen/lost reader to obtain sensitive information of any tagged object. Furthermore, Li et al. demonstrated that the server and the reader in this protocol do not authenticate each other. Besides, Li et al. pointed out that this protocol requires the server to perform an exhaustive search to validate a tag, which exhibits low efficiency in practical TIMSs. To remedy these weaknesses, Li et al. provided an enhanced version in [25]. Later in 2017, Benssalah et al. [26] illustrated that Li et al.’s protocol incurs traceability, impersonation and desynchronization attacks, and introduced an improvement. Unfortunately, Benssalah et al.’s protocol is still vulnerable to traceability and desynchronization attacks [27]. In 2018, Fan et al. [10] proposed an ultralightweight RFID authentication protocol, named LRMI, to protect medical privacy in IoT, using cross and rotation functions for authentication. Nevertheless, in 2019, Aghili et al. [28] analyzed the LRMI protocol and found that it cannot withstand traceability and impersonation attacks. Additionally, Aghili et al. proposed an improved version in [28], named SecLAP, which is based on modular rotation function. However, Safkhani et al. [29] discovered that the SecLAP protocol has a security vulnerability of secret disclosure, which allows an adversary to mount traceability and desynchronization attacks. Moreover, it is suggested that the ultralightweight operations such as the rotation, cross, and modular rotation functions do not converge to construct a secure protocol [29,30]. In the same year, Zhou et al. [31] presented a quadratic residue-based RFID authentication protocol for TIMSs. Later, Safkhani and Vasilakos [27] pointed out that Zhou et al.’s protocol [31] is prone to desynchronization attacks. They also proposed an improved protocol for TIMSs in [27]. In this improved protocol, the identifier of a tag is used as the secret key of the tag, which does not update so as to avoid desynchronization attacks. In the authentication phase, a tag encrypts its identifier with random numbers and timestamp using a hash function, and sends the ciphertext to the server for authentication. To verify the tag, the server needs to exhaust its database to find a tag identifier that can satisfy the received ciphertext. Thus, their protocol is inefficient. Besides, since the random numbers and timestamp are transmitted in plain text, once a tag identifier is exposed, an adversary can easily identify the tag’s messages in previous sessions, which implies that this protocol is destitute of forward secrecy.

Recently, Fan et al. proposed [14] a lightweight RFID authentication scheme based on quadratic residue theorem. The authors claimed that their scheme meets the security requirements necessary for RFID-based healthcare systems and is compatible with the EPC C1G2 standard. In this article, we demonstrate that this scheme has several security concerns.

## 3. Preliminary

### 3.1. Security Demands

An authentication scheme, which aims to secure a practical RFID-based healthcare system, should meet the following security demands.

(a) **Untraceability:** A tag should not be traced by an adversary. The adversary who stands between the tag and the reader may eavesdrop and correlate the tag’s messages from two different sessions so as to identify the tag.
(b) **Forward secrecy:** Even if the secret parameters of a tag are exposed to an adversary, the adversary can hardly identify the previous messages of the tag, which can be obtained by eavesdropping the read-tag channel.
(c) **Resilience to impersonation attacks:** An adversary may try to impersonate legitimate scheme parties (the server, reader, or tag), e.g., by replaying a message intercepted from the channels. Any impersonation should be prevented.
(d) **Resistance to desynchronization attacks:** If a scheme relies on shared values for authentication, an adversary may cause desynchronization problems. For example, if the server updates the shared values but the tag does not, the server may not be able to authenticate the tag in the future. Such desynchronization attacks should be resisted.
(e) **Scalability:** If the server needs to do an exhaustive search to verify a tag, the scheme is not scalable. Worse than that, an adversary may launch a time measurement attack [32] against the scheme, which can identify a tag according to its authentication time spent by the server. Thus, an authentication scheme should avoid any exhaustive search operation to ensure scalability.

### 3.2. Adversary Model

Researchers, who proposed the authentication schemes [10,14,25,26,27,28,31] for RFID-based healthcare systems in recent years, have a consensus that both the tag-to-reader channel and the reader-to-server channel are insecure so their security should be considered in the authentication schemes. Thus, we assume that an adversary can control both communication channels. The adversary is able to eavesdrop, modify, block, and replay the transferred messages. In addition, if the scheme leverages timestamps for authentication, we assume that the adversary can manipulate the time setting of the reader, which is practical for mobile readers [27].

We model the adversary A as a polynomial-time algorithm. Given a server, S, a reader, R, and a tag, T, the adversary A has access to the following oracles:

- Execute(S, R, T): A eavesdrops on both of the two communication channels during the execution of an instance of the scheme between T, R, and S. This oracle models the adversary’s ability to monitor the channels between scheme parties.
- Send(X, m_1_, m_2_): A sends a message m_1_ to a scheme party X and receives a message m_2_ from X. This oracle models the adversary’s ability to act as a scheme party.
- Block(.): A blocks any message of the scheme. This oracle models the adversary’s ability to stage a denial of service attack by jamming the communication channels.
- SetTime(R, t): A sets the current time of the reader R to time t. This oracle models the adversary’s ability to control the reader’s time setting.
- Reveal(T): A manages to obtain the secret parameters of the tag T. The oracle models the adversary’s ability to crack a tag and access its secrets.

The adversary A can invoke the oracles Execute, Send, Block, Time, and SetTime any polynomial number of times. However, the Reveal oracle can be called only once for each tag. If the tag is already compromised, it is meaningless to invoke the Reveal oracle on the same tag again.

### 3.3. Notations

The notations used for scheme description are presented in Table 1.

## 4. Review of Fan et al.’s Scheme

In this section, we first review Fan et al.’s scheme [14] and then perform a security analysis of this scheme.

### 4.1. Fan et al.’s Scheme

**Figure 2:**
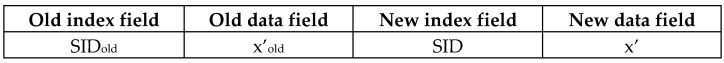
Tags’ index data table in Fan et al.’s scheme.

**Figure 3:**
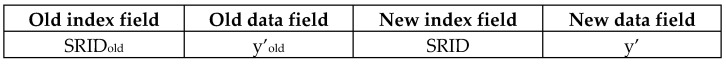
Readers’ index data table in Fan et al.’s scheme.

In Fan et al.’s scheme, as shown in Figure 2, the server stores the current pseudo identifier SID and secret data x’ of a tag in an index data table, in which the current pseudo identifier is used as an index. The old pseudo identifier and secret data of the tag are also recorded in the table. Similarly, the current pseudo identifier and secret data of a reader and also the old ones are stored in another index data table, as presented in Figure 3.

**Figure 4:**
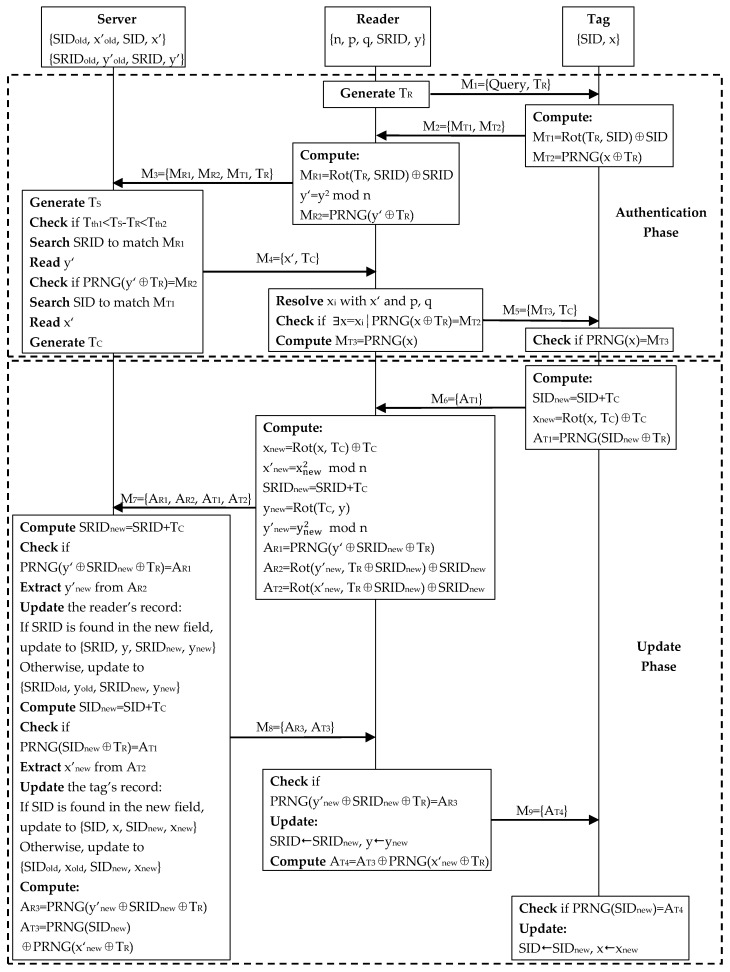
Authentication and update phases of Fan et al.’s scheme.

Fan et al.’s scheme consists of an initial phase, authentication phase, and update phase. The last two phases are demonstrated in Figure 4.

#### 4.1.1. Initial Phase

**Step** **1:** The system administrator generates two big primes p and q (the length of each is at least 512 bits), computes n = pq, and stores n, p, and q in each legitimate reader.
**Step** **2:** For each legitimate reader, the administrator assigns a pseudo identifier SRID and a secret key y. The length of y is at least 1024 bits. In the readers’ index data table stored in the server, the administrator sets SRID = SRID and y’ = y^2^ mod n while SRID_old_ and y’_old_ are both set to 0.
**Step** **3:** For each legitimate tag, the administrator assigns a pseudo identifier SID and a secret key x. The length of x is at least 1024 bits. In the tags’ index data table stored in the server, the administrator sets SID = SID and x’ = x^2^ mod n while SID_old_ and x’_old_ are both set to 0.

#### 4.1.2. Authentication Phase

**Step** **1:** Reader→Tag: M_1_ = {Query, T_R_}

The reader sends “Query” along with its current time T_R_ to the tag.

**Step** **2:** Tag→Reader: M_2_ = {M_T1_, M_T2_}

After receiving M_1_, the tag computes M_T1_ = Rot(T_R_, SID)⨁SID, M_T2_ = PRNG(x⨁T_R_), and sends {M_T1_, M_T2_} to the reader.

**Step** **3:** Reader→Server: M_3_ = {M_R1_, M_R2_, M_T1_, T_R_}

Upon receipt of M_2_, the reader computes M_R1_ = Rot(T_R_, SRID) ⨁SRID, y’ = y^2^ mod n, M_R2_ = PRNG(y’⨁T_R_), and sends {M_R1_, M_R2_, M_T1_, T_R_} to the server.

**Step** **4:** Server→Reader: M_4_ = {x‘, T_C_}

Once M_3_ is received, the server generates its current time T_S_ and checks whether T_th1_ < T_S_- T_R_ < T_th2_. If so, the server checks the records in the readers’ index data table to find an SRID for the matching M_R1_ = Rot(T_R_, SRID) ⨁SRID. If found, the server reads y’ from the corresponding record to check if PRNG(y’⨁T_R_) = M_R2_. If M_R2_ is correct, the reader is valid. Then, the server checks the records in the tags’ index data table to find a SID for the matching M_T1_ = Rot(T_R_, SID) ⨁SID. If there is a match, the server reads the corresponding x’ and sends it along with the server’s current time T_C_ to the reader.

**Step** **5:** Reader→Tag: M_5_ = {M_T3_, T_C_}

Upon receiving M_4_, the reader resolves four solutions x_1_, x_2_, x_3_, x_4_ with x’ and p, q. Then, it checks whether there exists a x = x_i_ (i = 1, 2, 3, 4) that can satisfy PRNG(x⨁T_R_) = M_T2_. If so, the tag is legitimate. The reader computes M_T3_ = PRNG(x) and sends {M_T3_, T_C_} to the tag.

**Step** **6:** Validation at the tag.

Once M_5_ arrives, the tag checks whether the value of M_T3_ is PRNG(x). If so, the reader is authenticated. The authentication phase ends here, followed by the update phase.

#### 4.1.3. Update Phase

**Step** **1:** Tag→Reader: M_6_ = {A_T1_}

The tag computes SID_new_ = SID + T_C_, x_new_ = Rot(x, T_C_) ⨁T_C_, A_T1_ = PRNG(SID_new_⨁T_R_), and sends A_T1_ to the reader.

**Step** **2:** Reader→Server: M_7_ = {A_R1_, A_R2_, A_T1_, A_T2_}

After the receipt of M_6_, the reader computes x_new_ = Rot(x, T_C_)⨁T_C_, x’_new_ = $x_{\mathrm{new}}^{2}$ mod n, SRID_new_ = SRID + T_C_, y_new_ = Rot(T_C_, y), y’_new =_ $y_{\mathrm{new}}^{2}$ mod n, A_R1_ = PRNG(y’⨁SRID_new_⨁T_R_), A_R2_ = Rot(y’_new_, T_R_⨁SRID_new_)⨁SRID_new_, A_T2_ = Rot(x’_new_, T_R_⨁SRID_new_)⨁SRID_new_, and sends {A_R1_, A_R2_, A_T1_, A_T2_} to the server.

**Step** **3:** Server→Reader: M_8_ = {A_R3_, A_T3_}

Once M_7_ is received, the server computes SRID_new_ = SRID + T_C_ to check whether PRNG(y’⨁SRID_new_⨁T_R_) = A_R1_. If so, the server extracts y’_new_ from A_R2_ and begins to update the reader’s record. If SRID is found in the new index field, the server lets SRID_old_←SRID, y’_old_←y‘, SRID←SRID_new_, y’←y’_new_. Otherwise, the server just lets SRID←SRID_new_, y’←y’_new_. Then, the server computes SID_new_ = SID + T_C_ to check whether PRNG(SID_new_⨁T_R_) = A_T1_. If so, the server extracts x’_new_ from A_T2_ and begins to update the tag’s record. If SID is found in the new index field, the server lets SID_old_←SID, x’_old_←x‘, SID←SID_new_, x’←x’_new_. Otherwise, the server just lets SID←SID_new_, x’←x’_new_. At last, the server computes A_R3_ = PRNG(y’_new_⨁SRID_new_⨁T_R_), A_T3_ = PRNG(SID_new_)⨁PRNG(x‘_new_⨁T_R_), and sends {A_R3_, A_T3_} to the reader.

**Step** **4:** Reader→Tag: M_9_ = {A_T4_}

Upon receiving M_8_, the reader checks whether PRNG(y’_new_⨁SRID_new_⨁T_R_) = A_R3_. If so, the reader updates SRID←SRID_new_, y←y_new_, computes A_T4_ = A_T3_⨁PRNG(x‘_new_⨁T_R_) and sends A_T4_ to the tag.

**Step** **5:** Validation at the tag.

After M_9_ arrives, the tag checks whether PRNG(SID_new_) = A_T4_. If so, the tag updates SID←SID_new_, x←x_new_.

### 4.2. Security Analysis of Fan et al.’s Scheme

Although Fan et al. claimed that their scheme is secure, we prove that this scheme cannot provide forward secrecy and is not resistant against impersonation attacks.

#### 4.2.1. Attack against Forward Secrecy

**Theorem** **1.** *Fan et al.’s scheme cannot ensure forward secrecy.*

**Proof.**  In Fan et al.’s scheme, if an adversary manages to obtain the current pseudo identifier SID and secret key x of a tag, the adversary can correlate the tag with its messages before completing the last scheme run with valid scheme parties. This is modeled by the following game between the challenger C as the RFID system and the adversary A. Assumed that both C and A have the power no more than a polynomial-time algorithm:

(1) C selects two tags, T_0_ and T_1_, a reader R, and a server S, which are all valid.
(2) A calls the oracles Execute, Send, and Block for a polynomial number of times on T_0_, T_1_, R, and S.
(3) A stops and notifies C.
(4) C randomly selects a bit b and sets T = T_b_
(5) A invokes the oracle Reveal(T).
(6) A outputs a bit b’. If b’ = b, A wins the game.

The advantage of successfully identifying the tag is defined as Adv_A_ = $2\times \left(\mathrm{Pr}\left[b^{\prime }=b\right]-\frac{1}{2}\right)$. If the adversary A has no advantage over the random guess, $\mathrm{Pr}\left[b^{\prime }=b\right]=\frac{1}{2}$. Thus, Fan et al.’s scheme fails to ensure forward secrecy if Adv_A_ > 0. For easy reading, we denote a parameter P in the i-th session of the tag T as PTi.

Suppose the challenger C selects two tags, T_0_ and T_1_, a reader R, and a server S for the game. A starts the game and calls the oracles Execute, Send, and Block for a polynomial number of times on T_0_, T_1_, R and S. Assume that C carries out a complete instance of the scheme, denoted as the i-th session, with each tag. After the i-th session is finished, the pseudo identifier of the tag T_j_ (j ∈ {0, 1}), STjiID, has been updated to STjiIDnew. A records the parameter A_T4_ in the i-th session of the tag T_0_, denoted as AT0iT4, and notifies C. Then, C chooses a random bit b and sets T = T_b_. Now, A calls the oracles Reveal(T) to obtain the current pseudo identifier of the tag T, denoted as _T_SID. Obviously, _T_SID is either ST0iIDnew or ST1iIDnew. Then, A computes PRNG(_T_SID). If PRNG(_T_SID) = AT0iT4, A outputs a bit b’ = 0 since AT0iT4 = PRNG(ST0iIDnew). Otherwise, A outputs a bit b’ = 1. Therefore, the probability that Pr(b’ = b) is 1. So the advantage of the adversary A in the tag identification, Adv_A_, is 1, which proves that Fan et al.’s scheme cannot provide forward secrecy. □

This security flaw is due to the fact that the value of A_T4_ is only related to the updated tag pseudo identifier SID_new_.

#### 4.2.2. Impersonation Attack

**Theorem** **2.** *In Fan et al.’s scheme, an adversary can impersonate as a legitimate reader to the tag.*

**Proof.**  In the authentication phase of Fan et al.’s scheme, a tag authenticates a reader through the message M_5_. To model the adversary’s attempt to impersonate as a legitimate reader to a tag, we use the following game between the challenger C and the adversary A.

(1) C chooses a tag T, a reader R, and a server S, which are all valid.
(2) A calls the oracles Execute, Send, and Block for a polynomial number of times on T, R, and S.
(3) A stops and notifies C.
(4) A invokes the Send oracle to impersonate as a reader.
(5) If A is authenticated by the tag T as a valid reader, A wins the game.

Suppose the challenger C selects a tag T, a reader R, and a server S for the game. A starts the game and calls the oracles Execute, Send, and Block for a polynomial number of times on T, R and S. Assume that C carries out an instance of the scheme on T, R and S. A records the messages M_1_ and M_5_, and blocks the message M_5_ so that the update phase does not execute. The message M_1_ consists of “Query” and T_R_. The message M_5_ consists of M_T3_ and T_C_. The value of M_T3_ is PRNG(x), in which x is the secret key of the tag T. Then, A notifies C. Now, A invokes the Send oracle to impersonate as a reader to T. Specifically, A sends the stored M_1_ to T and receives the response from T. After that, A sends the stored M_5_ to T. Upon receipt of the stored M_5_, T checks whether PRNG(x) = M_T3_. Since T’s secret key x does not update in the last scheme run, the condition satisfies. Thus, the adversary A wins the game with a probability of 1. So, an adversary can impersonate as a legitimate reader to the tag in Fan et al.’s scheme. □

The reason for this security flaw is that the authentication parameter M_T3_ contains no randomness produced by the tag.

**Theorem** **3.** *In Fan et al.’s scheme, an adversary can impersonate as a legitimate reader to the server.*

**Proof.**  In the authentication phase of Fan et al.’s scheme, a server authenticates a reader through the message M_3_. If an adversary has the ability to manipulate the time setting of the reader, as the adversary model explained in Section 3.2, the adversary is able to impersonate as a legitimate reader to the server. The impersonation attempt is modeled as the following game between the challenger C and the adversary A.

(1) C chooses a tag T, a reader R, and a server S, which are all valid.
(2) A calls the oracles Execute, Send, Block, and SetTime for a polynomial number of times on T, R, and S.
(3) A stops and notifies C.
(4) A invokes the Send oracle to impersonate as a reader.
(5) If A is authenticated by the server S as a valid reader, A wins the game.

Suppose the challenger C selects a tag T, a reader R, and a server S for the game. A starts the game and calls the oracles Execute, Send, Block, and SetTime for a polynomial number of times on T, R and S. Specifically, A changes the time of the reader R to a future time t_1_. Assume that C immediately carries out an instance of the scheme on T, R and S. In this session, the reader R sends M_1_ = {Query, t_1_} to the tag T. Upon receiving M_1_, the tag T computes M_T1_ = Rot(t_1_, SID)⨁SID, M_T2_ = PRNG(x⨁t_1_), and sends M_2_ = {M_T1_, M_T2_} to the reader. After M_2_ arrives, the reader computes M_R1_ = Rot(t_1_, SRID)⨁SRID, y’ = y^2^ mod n, M_R2_ = PRNG(y‘⨁t_1_), and sends M_3_ = {M_R1_, M_R2_, M_T1_, t_1_} to the server. A records the messages M_1_ and M_3_, and blocks the messages M_3_.

Then, A sets the time of the reader R to the correct time to synchronize with the time of the server S. Before the time t_1_, A blocks any message sent to the server S so that no updates will be done. At the time t_1_, A notifies C and invokes the Send oracle to impersonate as a reader to S. Specifically, A sends the stored M_1_ to the tag T. Upon receipt of the response from T, A sends the stored M_3_ to S. Once the stored M_3_ is received, S generates a timestamp T_S_ and checks whether T_th1_ < T_S_ – t_1_ < T_th2_. Because A starts the current session at the time t_1_, A can pass the check. After that, S searches the readers’ index data table to find an SRID for the matching Rot(t_1_, SRID)⨁SRID = M_R1_. Since the reader pseudo identifier SRID does not update, there is a match. Then, S checks whether PRNG(y‘⨁t_1_) = M_R2_. Because the reader’s secret data y’ does not update, the condition satisfies. In this way, the adversary A is authenticated as a valid reader by the server with a probability of 1. Therefore, an adversary can impersonate as a legitimate reader to the server in Fan et al.’s scheme. □

This security flaw is because that T_R_ is the current time of the reader R. By manipulating the reader’s time, an adversary can obtain the parameters, M_R1_ and M_R2_, related to a future time.

## 5. The Proposed Scheme

In this section, we first propose an improved scheme to overcome the security vulnerabilities of Fan et al.’s scheme [14]. Moreover, to satisfy the EPC C1G2 standard and the mobile environment in an RFID-based healthcare system, the heavyweight cryptographic primitives should not be used. In the proposed scheme, we just leverage the operations supported by an EPC C1G2 tag to secure both the reader-tag channel and the server-reader channel. Although it is feasible to adopt a mutual authenticated TLS channel between the server and the reader to secure the server-reader channel, our scheme can just use the lightweight operations to achieve the same goal with lower overhead. We also formally analyze our proposed scheme on the major security demands.

### 5.1. Scheme Description

**Figure 5:**
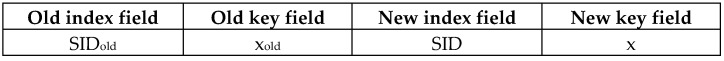
Tags’ index data table in our proposed scheme.

**Figure 6:**
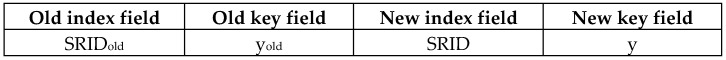
Readers’ index data table in our proposed scheme.

As shown in Figure 5, the server stores the current pseudo identifier SID and secret key x of a tag in an index data table. The current pseudo identifier is used as an index in the table. The previous index SID_old_ and secret key x_old_ of the tag are also recorded in the table to prevent desynchronization attacks. Similarly, the current pseudo identifier SRID and secret key y of a reader are stored in another index data table and so are the previous ones, as demonstrated in Figure 6. Our proposed scheme includes an initial phase and an authentication phase.

#### 5.1.1. Initial Phase

**Step** **1:** For each legitimate tag, the administrator assigns a pseudo identifier SID and a secret key x. The administrator then sets SID = SID and x = x in the tags’ index data table while SID_old_ and x_old_ are both set to 0.
**Step** **2:** For each legitimate reader, the administrator assigns a pseudo identifier SRID and a secret key y. The administrator then sets SRID = SRID and y = y in the readers’ index data table while SRID_old_ and y_old_ are both set to 0.

#### 5.1.2. Authentication Phase

**Figure 7:**
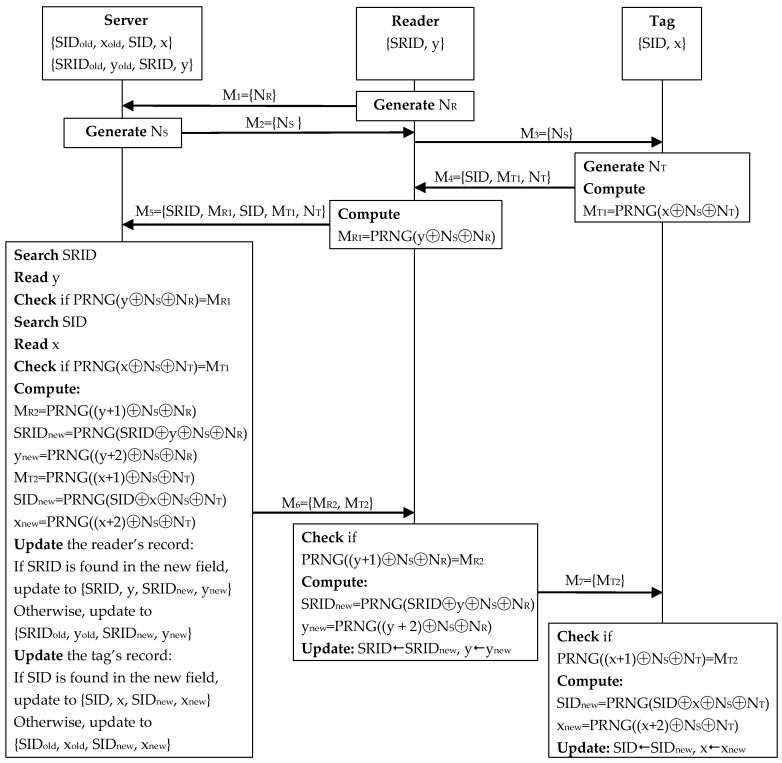
Authentication phase of our improved scheme.

The authentication phase of our proposed scheme is presented in Figure 7. This phase consists of the following steps:

**Step** **1:** Reader→Server: M_1_ = {N_R_}

The reader generates a random number N_R_ and sends it to the server.

**Step** **2:** Server→Reader: M_2_ = {N_S_}

After receiving M_1_, the server generates a random number N_T_ and sends it to the reader.

**Step** **3:** Reader→Tag: M_3_ = {N_S_}

Upon receipt of M_2_, the reader forwards N_S_ to the tag.

**Step** **4:** Tag→Reader: M_4_ = {SID, M_T1_, N_T_}

Once M_3_ is received, the tag generates a random number N_T_, computes M_T1_ = PRNG(y⨁N_S_⨁N_T_), and sends {SID, M_T1_, N_T_} to the reader.

**Step** **5:** Reader→Server: M_5_ = {SRID, M_R1_, SID, M_T1_, N_T_}

After M_4_ arrives, the reader computes M_R1_ = PRNG(y⨁N_S_⨁N_R_) and composes a reply {SRID, M_R1_, SID, M_T1_, N_T_} to the server.

**Step** **6:** Server→Reader: M_6_ = {M_R2_, M_T2_}

Upon receiving M_5_, the server searches for the received SRID in the readers’ index data table. If found, the server reads the corresponding y to check whether PRNG(y⨁N_S_⨁N_R_) = M_R1_. If so, the reader is valid. Then, the server searches for the received SID in the tags’ index data table. If found, the server reads the corresponding x to check whether PRNG(x⨁N_S_⨁N_T_) = M_T1_. If so, the tag is valid.

After confirming the validity of both the reader and tag, the server computes M_R2_ = PRNG((y + 1)⨁N_S_⨁N_R_), SRID_new_ = PRNG(SRID⨁y⨁N_S_⨁N_R_), y_new_ = PRNG((y + 2)⨁N_S_⨁N_R_), M_T2_ = PRNG((x + 1)⨁N_S_⨁N_T_), SID_new_ = PRNG(SID⨁x⨁N_S_⨁N_T_), and x_new_ = PRNG((x + 2)⨁N_S_⨁N_T_). Then, the server updates the readers’ index data table. If SRID is found in the new index field, the server lets SRID_old_←SRID, y_old_←y, SRID←SRID_new_, y←y_new_. Otherwise, the server just lets SRID←SRID_new_, y←y_new_. Similarly, the server updates the tags’ index data table. If SID is found in the new index field, the server lets SID_old_←SID, x_old_←x, SID←SID_new_, x←x_new_. Otherwise, the server just lets SID←SID_new_, x←x_new_. Once the updating is finished, the server sends {M_R2_, M_T2_} to the reader.

**Step** **7:** Reader→Tag: M_7_ = {M_T2_}

After M_6_ is received, the reader checks whether PRNG((y + 1)⨁N_S_⨁N_R_) = M_R2_. If so, the server is valid and has updated the readers’ index data table. Since the server sends out M_R2_ only when the tag is legitimate, the reader authenticates the tag implicitly via M_R2_. Then, the reader computes SRID_new_ = PRNG(SRID⨁y⨁N_S_⨁N_R_), y_new_ = PRNG((y + 2)⨁N_S_⨁N_R_), and updates SRID←SRID_new_, y←y_new_. After that, the reader sends M_T2_ to the tag.

**Step** **8:** Validation at the tag.

Once M_7_ arrives, the tag checks whether PRNG((x + 1)⨁N_S_⨁N_T_) = M_T2_. If so, the server is valid and has updated the tags’ index data table. The tag also implicitly authenticates the reader since the tag will not receive a valid M_T2_ unless the server has authenticated the reader. Then, the tag computes SID_new_ = PRNG(SID⨁x⨁N_S_⨁N_T_), x_new_ = PRNG((x + 2)⨁N_S_⨁N_T_) and updates SID←SID_new_, x←x_new_.

### 5.2. Security Analysis

**Lemma** **1.** *In the proposed scheme, the secret keys cannot be exposed without calling the Reveal oracle.*

**Proof.**  In the scheme, the transferred parameters related to the tag secret key x include M_T1_ and M_T2_, which are generated by M_T1_ = PRNG(x⨁N_S_⨁N_T_) and M_T2_ = PRNG((x+1)⨁N_S_⨁N_T_), respectively. An adversary cannot obtain x from M_T1_ or M_T2_ because PRNG() is regarded as an irreversible operation [14]. On the other hand, the transferred parameters related to the reader secret key y include M_R1_ and M_R2_, which are generated by M_R1_ = PRNG(y⨁N_S_⨁N_R_) and M_R2_ = PRNG((y+1)⨁N_S_⨁N_R_), respectively. Since PRNG() is irreversible, the adversary cannot get y from M_R1_ or M_R2_. Therefore, unless the adversary calls the Reveal oracle, the secret keys cannot be revealed. □

**Lemma** **2.** *In the proposed scheme, two of the message parameters, before and after completing a scheme run with valid scheme parties, cannot be correlated without calling the Reveal oracle.*

**Proof.**  For easy reading, we denote a parameter P in the i-th session as ^i^P. Without loss of generality, we assume that an adversary attempts to correlate ^i^P with ^i+1^P. In our proposed scheme, the messages consist of nine parameters: N_S_, N_R_, N_T_, SID, SRID, M_T1_, M_T2_, M_R1_, and M_R2_.

First, we consider the parameters N_S_, N_R_, and N_T_. N_S_ is a random number generated in each session so the adversary cannot correlate ^i^N_S_ with ^i+1^N_S_. For the same reason, ^i^N_R_ and ^i^N_T_ cannot be correlated with ^i+1^N_R_ and ^i+1^N_T_, respectively.

Second, we consider the pseudo identifiers, SID and SRID. The value of ^i+1^SID is PRNG(^i^SID⨁^i^x⨁^i^N_S_⨁^i^N_T_). By Lemma 1, the adversary cannot obtain ^i^x. Thus, it is difficult for the adversary to correlate ^i^SID with ^i+1^SID unless the Reveal oracle is invoked. Similarly, the value of ^i+1^SRID is PRNG(^i^SRID⨁^i^y⨁^i^N_S_⨁^i^N_R_). Because ^i^y is not exposed, the adversary cannot correlate ^i+1^SRID with ^i^SRID.

Finally, we consider the remaining parameters. Since ^i^M_T1_ = PRNG(^i^x⨁^i^N_S_⨁^i^N_T_), ^i+1^x = PRNG((^i^x + 2 )⨁^i^N_S_⨁^i^N_T_) and ^i+1^M_T1_ = PRNG(^i+1^x⨁^i+1^N_S_⨁^i+1^N_T_) to correlate ^i^M_T1_ with ^i+1^M_T1_, the adversary needs to know ^i^x, which cannot be obtained without the Reveal oracle (by Lemma 1). For the same reason, ^i^M_T2_, whose value is PRNG((^i^x + 1)⨁^i^N_S_⨁^i^N_T_), cannot be correlated with ^i+1^M_T2_, whose value is PRNG((^i+1^x+1)⨁^i+1^N_S_⨁^i+1^N_T_). Similarly, since ^i^M_R1_ = PRNG(^i^y⨁^i^N_S_⨁^i^N_R_), ^i+1^y = PRNG((^i^y + 2)⨁^i^N_S_⨁^i^N_R_) and ^i+1^M_R1_ = PRNG(^i+1^y⨁^i+1^N_S_⨁^i+1^N_R_), without the knowledge of ^i^y, the adversary cannot correlate ^i^M_R1_ with ^i+1^M_R1_. For the same reason, ^i^M_R2_, whose value is PRNG(^i^y + 1⨁^i^N_S_⨁^i^N_R_), cannot be correlated with ^i+1^M_R2_, whose value is PRNG((^i+1^y + 1)⨁^i+1^N_S_⨁^i+1^N_R_).

Thus, without calling the Reveal oracle, the adversary cannot correlate two of the message parameters that are separated by a complete scheme run with valid scheme parties. □

**Theorem** **4.** *In the proposed scheme, tags are universally untraceable.*

**Proof.**  In an RFID scheme, a tag is universally untraceable [33] if an adversary cannot correlate two of the messages sent and received by the tag, separated by a complete scheme run with valid scheme parties. This is modeled by a game between the challenger C as the RFID system and the adversary A. Assumed that both C and A have the power no more than a polynomial-time algorithm:

(1) C selects two tags, T_0_ and T_1_, a reader R, and a server S, which are all valid.
(2) A calls the oracles Execute, Send, and Block for a polynomial number of times on T_0_, T_1_, R and S.
(3) A stops and notifies C.
(4) C randomly selects a bit b and sets T = T_b_.
(5) A calls the oracles Execute, Send, and Block on T, R and S.
(6) A outputs a bit b’. If b’ = b, A wins the game.

The advantage of successful tag identification is defined as Adv_A_ = $2\times \left(\mathrm{Pr}\left[b^{\prime }=b\right]-\frac{1}{2}\right)$. If the adversary A has no advantage over the random guess, $\mathrm{Pr}\left[b^{\prime }=b\right]=\frac{1}{2}$. Thus, tags are universally untraceable if Adv_A_ is 0.

Suppose the challenger C selects two tags, T_0_ and T_1_, a reader R, and a server S for the game. A starts the game and calls the oracles Execute, Send, and Block for a polynomial number of times on T_0_, T_1_, R and S. Assume that C carries out a complete instance of the scheme, denoted as the i-th session, with each tag. A records all the outputs of the oracle calls and notifies C. Then, C chooses a random bit b and sets T = T_b_. Now, A calls the oracles Execute, Send, and Block on T, R and S. Assume that C carries out a complete instance of the scheme with the tag T, denoted as the i+1-th session. A records all the outputs of the oracle calls and produces a guess bit b’. In the proposed scheme, the tag sends and receives the messages M_1_, M_2_, and M_7_, which consist of the following message parameters: SID, N_T_, N_R_, M_T1_, and M_T2_. Since A cannot correlate any message parameter in the i-th session with the parameter in the i+1-th session (by Lemma 2), A can only perform a random guess. Therefore, the probability that Pr[b’ = b] is $\frac{1}{2}$ and Adv_A_ is 0. So the tags in our proposed scheme are universally untraceable. □

**Theorem** **5.** *The proposed scheme can ensure forward secrecy.*

**Proof.**  We model this as the game in the proof of Theorem 1. The challenger C selects two tags, T_0_ and T_1_, a reader R, and a server S for the game. The adversary A starts the game and calls the oracles Execute, Send, and Block on T_0_, T_1_, R, and S for a polynomial number of times. Assume C carries out a complete instance of the scheme with each tag. A records the outputs of the oracle calls. Then, C generates a random bit b and sets T = T_b_. Hereafter, A calls the oracles Reveal(T) to obtain the pseudo identifier and secret key of the tag T. Finally, A outputs a guess bit b’.

Because the current secret key of T is generated from the PRNG of the previous one, A cannot inverse the PRNG function to obtain the previous secret key. Similarly, since the current pseudo identifier of T is generated from the PRNG of the previous one, A cannot deduce the previous pseudo identifier. Besides, by Lemma 2, A cannot correlate the previous pseudo identifier of T, which is either that of T_0_ or that of T_1_, with the current pseudo identifier of T. Therefore, A has no advantage over a random guess, which means that the proposed scheme can ensure forward secrecy. □

**Theorem** **6.** *The proposed scheme can resist impersonation attacks.*

**Proof.**  An adversary may attempt to impersonate as a tag, a reader or a server. We discuss these three cases as follows.

(a) Tag impersonation

We model this as the following game between the challenger C and the adversary A.

(1) C chooses a tag T, a reader R, and a server S, which are all valid.
(2) A calls the oracles Execute, Send, and Block for a polynomial number of times on T, R, and S.
(3) A stops and notifies C.
(4) A invokes the Send oracle to impersonate as a tag.
(5) If A is authenticated as a valid tag, A wins the game.

Suppose the challenger C selects a tag T, a reader R, and a server S for the game. A starts the game and calls the oracles Execute, Send, and Block for a polynomial number of times on T, R, and S. Assume that C carries out an instance of the scheme on T, R, and S. A records all the oracle outputs.

To pass the authentication, A must send a valid SID and a valid M_T1_ = PRNG(x⨁N_S_⨁N_T_). To do so, A needs to know the tag secret key x. However, by Lemma 1, A cannot obtain x to generate a valid M_T1_. On the other hand, assume that A calls the Block oracle to block the message M_5_ so that no updates will happen, and then notifies C. Hereafter, C carries out a new instance of the scheme on T, R, and S. To impersonate as a tag, A invokes the Send oracle to send the recorded SID, M_T1_, and N_T_ to the reader R as the response M_2_. However, since the reader R generates a new N_R_ in this scheme run, the recorded M_T1_ cannot be valid unless the new N_R_ happens to be the same as the old N_R_, whose probability is negligible. Therefore, A can hardly impersonate as a valid tag.

(b) Reader impersonation

Firstly, we consider that the adversary A attempts to impersonate as a valid reader to the tag. The attempt is modeled as the game in the proof of Theorem 2. To be validated by the tag T, A needs to send a valid M_T2_ = PRNG((x + 1)⨁N_S_⨁N_T_). However, by Lemma 1, A cannot obtain x to generate a valid M_T2_. On the other hand, assume that A blocks M_7_ to prevent any updating on the tag, and then notifies C. Hereafter, C carries out a new instance of the scheme on T, R, and S. To impersonate as a reader to the tag, A sends the recorded M_T2_ to the tag T. However, the recorded M_T2_ cannot be valid unless the old N_T_ is the same as the N_T_ generated in the new scheme run, which has a negligible probability.

Secondly, we consider that A tries to impersonate as a valid reader to the server, which can be modeled as a game similar to the one in the proof of Theorem 2, except that in the last step the adversary A should be authenticated by the server S. To be authenticated, A must send a valid SRID and a valid M_R1_ = PRNG(y⨁N_S_⨁N_R_) to the server. By Lemma 1, the reader secret key y is not exposed so A cannot generate a valid M_R1_. On the other hand, assume that A blocks M_5_ to prevent any updating, and then notifies C. Hereafter, C carries out a new instance of the scheme on T, R, and S. To impersonate as a reader to the server, A sends the recorded N_R_, SRID and M_R1_ to the server S. Since S generates a new N_S_ in the new scheme run, the recorded M_R1_ has a negligible probability to be valid.

Therefore, the probability to impersonate as a valid reader is negligible.

(c) Server impersonation

We model this attempt as a game similar to the one in the case (a), except that A calls the Send oracle to impersonate as a valid server. To impersonate as a legitimate server, A must send a valid M_R2_ = PRNG((y + 1)⨁N_S_⨁N_R_). However, without the knowledge of y (by Lemma 1), A fails to generate a valid M_R2_. On the other hand, assume that A blocks M_6_ to prevent any updating on the reader and tag, and then notifies C. Hereafter, C carries out a new instance of the scheme on T, R, and S. To impersonate as a server, A sends the recorded M_R2_ to the reader R. Because the new N_R_ is hardly the same as the old N_R_, the probability that the recorded M_R2_ can pass the authentication is negligible. Thus, the adversary A can impersonate as a valid server with a negligible probability.

In summary, the proposed scheme can defend against impersonation attacks. □

**Theorem** **7.** *The proposed scheme can ensure the resistance of desynchronization attacks.*

**Proof.**  In the proposed scheme, the server updates the index data tables after the message M_5_ is received and verified. If the message M_6_ is blocked, the reader does not update its pseudo identifier SRID and secret key y. Since SRID and y are stored in the old fields, the server can synchronize with the reader based on them. Assume that there is a new session and M_6_ is blocked again. In this session, since the server finds the received SRID in the old index field, the old values do not update. Thus, the server can still synchronize with the reader. Similarly, if M_6_ (or M_7_) is blocked, the server and tag can keep synchronization between them. On the other hand, as discussed in the proof of Theorem 6, an adversary cannot forge valid M_T1_ and M_R1_ to force the server to update the index data tables. Therefore, the proposed scheme is resistant to desynchronization attacks. □

**Theorem** **8.** *The proposed scheme is scalable.*

**Proof.**  According to Burmester et al. [34], if the server can find the record of a tag just based on the received data, the time cost can be constant. Otherwise, if some computation operations are needed before checking each record, an exhaustive search operation is needed to authenticate a tag, which results in time measurement attacks [32]. In the proposed scheme, the tag pseudo identifier is used as the index of the tags’ index data table so the server can find the tag’s record just by the received SID. Similarly, with the received SRID, the server can find the reader’s record. So the proposed scheme requires no exhaustive search operation. Therefore, the proposed scheme is of scalability and can also resist time measurement attacks. □

### 5.3. Formal Security Analysis with BAN-Logic

In this part, we employ BAN-logic [35] to perform a formal security analysis of our proposed scheme. The notations of BAN-logic are demonstrated in Table 2.

Then, we present the BAN-logic rules used in the analysis as below.

- **R1 (Seeing rule):**$\frac{P⊲\left\{X,Y\right\}}{P⊲X}$, it means when P receives a message set {X, Y}, P receives the message X.

- **R2 (Message-meaning rule):**$\frac{\left(P|\equiv P\overset{K}{↔}Q,P⊲{\left\{X\right\}}_{K}\right)}{P\left|\equiv Q\right|\sim X}$, it means if P believes that P and Q have a shared key K, P receives a message X encrypted by K, which indicates P believes Q has sent X to P.

- **R3 (Freshness rule):**$\frac{P|\equiv \#X}{P|\equiv \#\left\{X,Y\right\}}$, it means if P believes the message X is fresh, P believes the message set {X, Y} is fresh.

- **R4 (Nonce-verification rule):**$\frac{\left(P\left|\equiv \#X,P\right|\equiv Q|\sim X\right)}{P\left|\equiv Q\right|\equiv X}$, it means if P believes X is fresh, and Q has sent X, which indicates P believes Q believes X.

In the following analysis, the server, reader, and tag are denoted by S, R, and T, respectively.

#### 5.3.1. Idealized Form

Based on the BAN-logic notations, the message transmissions of our proposed scheme are idealized as below.

- **IM1:** $S⊲N_{R}$

- **IM2:** $R⊲N_{S}$

- **IM3:** $T⊲N_{S}$

- **IM4:** $R⊲\mathrm{SID},\;{\left\{\mathrm{PRNG}\left(x⨁N_{S}⨁N_{T}\right)\right\}}_{x},{\;N}_{T}$

- **IM5:** $S⊲\mathrm{SRID},\;{\left\{\mathrm{PRNG}\left(y⨁N_{S}⨁N_{R}\right)\right\}}_{y},\;\mathrm{SID},\;{\left\{\mathrm{PRNG}\left(x⨁N_{S}⨁N_{T}\right)\right\}}_{x},{\;N}_{T}$

- **IM6:** $R⊲{\left\{\mathrm{PRNG}\left(\left(y+1\right)⨁N_{S}⨁N_{R}\right)\right\}}_{y}$

, ${\left\{\mathrm{PRNG}\left(\left(x+1\right)⨁N_{S}⨁N_{T}\right)\right\}}_{x}$

- **IM7:** $T⊲{\left\{\mathrm{PRNG}\left(\left(x+1\right)⨁N_{S}⨁N_{T}\right)\right\}}_{x}$

#### 5.3.2. Initial Assumptions

The initial assumptions of our proposed scheme are as follows, specifying the initial process and belief of data.

- **A1:** $T|\equiv T\overset{x}{↔}S,S|\equiv T\overset{x}{↔}S$

- **A2:** $R|\equiv R\overset{y}{↔}S,S|\equiv R\overset{y}{↔}S$

- **A3:** $T|\equiv \#\left(N_{T}\right)$

- **A4:** $R|\equiv \#\left(N_{R}\right)$

- **A5:** $S|\equiv \#\left(N_{S}\right)$

#### 5.3.3. Security Goals

Since our proposed scheme aims to achieve mutual authentication between the genuine scheme parties, the security goals of should be achieved are listed as follows.

- **G1:** $S|\equiv R|\equiv {\left\{\mathrm{PRNG}\left(y⨁N_{S}⨁N_{R}\right)\right\}}_{y}$

- **G2:** $R|\equiv S|\equiv {\left\{\mathrm{PRNG}\left(\left(y+1\right)⨁N_{S}⨁N_{R}\right)\right\}}_{y}$

- **G3:** $S|\equiv T|\equiv {\left\{\mathrm{PRNG}\left(x⨁N_{S}⨁N_{T}\right)\right\}}_{x}$

- **G4:** $T|\equiv S|\equiv {\left\{\mathrm{PRNG}\left(\left(x+1\right)⨁N_{S}⨁N_{T}\right)\right\}}_{x}$

#### 5.3.4. Security Proofs

In this part, we prove the security goals of our scheme.

- **G1:** $S|\equiv R|\equiv {\left\{\mathrm{PRNG}\left(y⨁N_{S}⨁N_{R}\right)\right\}}_{y}$

**Proof.**  By IM5 and R1, we have

$$S⊲{\left\{\mathrm{PRNG}\left(y⨁N_{S}⨁N_{R}\right)\right\}}_{y}\;(E1)$$

Given E1, A2, and R2, we obtain

$$S|\equiv R\sim {\left\{\mathrm{PRNG}\left(y⨁N_{S}⨁N_{R}\right)\right\}}_{y}\;(E2)$$

In accordance with A5 and R3, we get

$$S|\equiv \#{\left\{\mathrm{PRNG}\left(y⨁N_{S}⨁N_{R}\right)\right\}}_{y}\;(E3)$$

With E2, E3 and R4, we can deduce $S|\equiv R|\equiv {\left\{\mathrm{PRNG}\left(y⨁N_{S}⨁N_{R}\right)\right\}}_{y}$. Therefore, G1 is proved. □

- **G2:** $R|\equiv S|\equiv {\left\{\mathrm{PRNG}\left(\left(y+1\right)⨁N_{S}⨁N_{R}\right)\right\}}_{y}$

**Proof.**  Based on IM6 and R1, we get

$$R⊲{\left\{\mathrm{PRNG}\left(\left(y+1\right)⨁N_{S}⨁N_{R}\right)\right\}}_{y}\;(E4)$$

With E4, A2, and R2, we know

$$R|\equiv S\sim {\left\{\mathrm{PRNG}\left(\left(y+1\right)⨁N_{S}⨁N_{R}\right)\right\}}_{y}\;(E5)$$

Given A4 and R3, we have

$$R|\equiv \#{\left\{\mathrm{PRNG}\left(\left(y+1\right)⨁N_{S}⨁N_{R}\right)\right\}}_{y}\;(E6)$$

Taking into account E5, E6, and R4, we can prove $R|\equiv S|\equiv {\left\{\mathrm{PRNG}\left(\left(y+1\right)⨁N_{S}⨁N_{R}\right)\right\}}_{y}$. Thus, G2 is achieved. □

- **G3:** $S|\equiv T|\equiv {\left\{\mathrm{PRNG}\left(x⨁N_{S}⨁N_{T}\right)\right\}}_{x}$

**Proof.**  According to IM5 and R1, we obtain

$$S⊲{\left\{\mathrm{PRNG}\left(x⨁N_{S}⨁N_{T}\right)\right\}}_{x}\;(E7)$$

By E7, A1, and R2, we have

$$S|\equiv T\sim {\left\{\mathrm{PRNG}\left(x⨁N_{S}⨁N_{T}\right)\right\}}_{x}\;(E8)$$

On the basis of A5 and R3, we get

$$S|\equiv \#{\left\{\mathrm{PRNG}\left(x⨁N_{S}⨁N_{T}\right)\right\}}_{x}\;(E9)$$

With E8, E9, and R4, we can deduce $S|\equiv T|\equiv {\left\{\mathrm{PRNG}\left(x⨁N_{S}⨁N_{T}\right)\right\}}_{x}$. Therefore, G3 is proved. □

- **G4:** $T|\equiv S|\equiv {\left\{\mathrm{PRNG}\left(\left(x+1\right)⨁N_{S}⨁N_{T}\right)\right\}}_{x}$

**Proof.**  In accordance with IM7, A1, and R2, we get

$$T|\equiv S\sim {\left\{\mathrm{PRNG}\left(\left(x+1\right)⨁N_{S}⨁N_{T}\right)\right\}}_{x}\;(E10)$$

Taking into account A3 and R3, we obtain

$$T|\equiv \#{\left\{\mathrm{PRNG}\left(\left(x+1\right)⨁N_{S}⨁N_{T}\right)\right\}}_{x}\;(E11)$$

Based on E10, E11, and R4, we can prove $T|\equiv S|\equiv {\left\{\mathrm{PRNG}\left(\left(x+1\right)⨁N_{S}⨁N_{T}\right)\right\}}_{x}$. Thus, G4 is achieved. □

Since all security goals are verified, our proposed scheme satisfies the logic security.

## 6. Performance Evaluation

In this section, we analyze the performance of our proposed scheme by comparing it with some recent schemes (published since 2018) [10,14,27,28,31] for RFID-based healthcare systems.

### 6.1. Security Performance

We compare the performance of our proposed scheme based on the security demands essential for RFID-based healthcare systems as demonstrated in Table 3. In the table, the symbol “Yes” represents that the scheme meets a security demand while the symbol “No” denotes that the scheme fails to satisfy a security demand. From Table 3, we can see that only our proposed scheme can guarantee all the desired security demands while other schemes fail to meet one or more security demands. As presented in Section 4.2, Fan et al.’s scheme [14] cannot support forward secrecy and is vulnerable to impersonation attacks. The security of other existing schemes has been discussed in Section 2. Safkhani and Vasilakos’s scheme [27] fails to ensure forward secrecy and scalability. The LRMI scheme [10] cannot resist traceability and impersonation attacks. The SecLAP scheme [28] is prone to traceability and desynchronization attacks. Zhou et al.’s scheme [31] is unable to withstand desynchronization attacks. The security of our proposed scheme has been analyzed in Section 5.2 and Section 5.3.

### 6.2. Efficiency Performance

We also compare the performance of our proposed scheme with other schemes in terms of costs for computation, communication, storage, and hardware implementation.

Firstly, the performance comparison in terms of the computation cost is presented in Table 4. We ignore simple operations such as concatenation, exclusive-OR, and addition. Table 4 shows the number of operations including rotation (denoted as Rot), the inverse operation of rotation (denoted as Rot^−1^), pseudo random number generation (denoted as P), hash (denoted as H), cross (denoted as C), modular rotation (denoted as MR) and squaring root solving operation (denoted as SR), which are required by our scheme and other schemes. From the column “Tag” of Table 4, we can notice that our proposed scheme only needs a tag to perform the pseudo random number generation operation, a preset operation for EPC C1G2 tags, while other schemes require a tag to perform some operations not implemented by EPC C1G2 tags. Thus, our proposed scheme has the best compatibility with the EPC C1G2 standard.

According to the experiment results in Section 4.3 of Zhou et al. [31], the time costs of hash, pseudo random number generation, modular squaring, and squaring root solving operations are 0.253, 0.021, 1.896, and 3.481 ms, respectively. As the cross, rotation, and modular rotation are ultralightweight operations, their time cost is negligible in computation. With these data, we can estimate the computation cost of each scheme, as illustrated in Table 5. From Table 5, we can see that the computation cost of our scheme is just higher than the ultralightweight schemes [10,28]. However, it can be justified since our scheme offers a higher security level than all other schemes.

Secondly, we compare the efficiency of our proposed scheme to other schemes in terms of the communication and storage cost. Since RFID tags have limited storage capacity while readers and servers have relatively sufficient storage capacity, the storage cost comparison focuses on the tag’s costs. For the schemes not based on quadratic residues, we assume that the lengths of parameters such as identifiers, secret keys, random numbers, timestamps, and function outputs are all L bits. For the quadratic residue-based schemes, we assume that the length of a secret key is L_QK_ bits and the length of the output of modulo squaring operation is L_MS_ bits while other parameters have the same length of L bits. L_QK_ and L_MS_ are usually greater than L for security purposes. According to Fan et al. [14], L_QK_ and L_MS_ are suggested to be at least 1024 while the length of a common tag EPC, used as a tag’s identifier, is 96 bits. Thus, for an intuitive comparison of the communication cost and storage cost, we assume L_QK_ = L_MS_ = 1024 while L = 96. Besides, to be fair, we omit the cost of the string “Query” since most schemes do not use it. The comparison results are demonstrated in Table 6.

In our proposed scheme, there are seven transferred messages consisting of fourteen 96-bit parameters, which results in a communication cost of 1344 bits. From Table 6, we can see that our scheme and the scheme in [27] have the same communication cost, which is less than the rest of the schemes. Besides, a tag in our scheme needs to store an identifier and a secret key, leading to a storage cost of 192 bits. Table 6 shows that the storage cost of our scheme is just higher than that of the scheme in [27] because a tag stores only an identifier in this scheme. However, the scheme in [27] is less secure than our scheme.

Finally, we discuss the hardware implementation cost. Considering that the server and the reader have much more resources than the tag, we focus on the implementation cost imposed on the tag. From the “Tag” column of Table 4, we can know that the security primitives, used by tags in our scheme and other schemes, include rotation function, cross function, modular rotation function, pseudo random number generator (PRNG), hash function, and modular squaring function. The authors in [10,14,28] present the FPGA implementation costs of the rotation, cross, and modular rotation functions, which are 112, 1, and 65 lookup tables (LUTs), respectively. Due to the limited resource on a tag, lightweight PRNG and hash function should be adopted. For instance, Mandal et al. [36] designed a lightweight PRNG satisfying the EPC C1G2 standard, named Warbler, which can be implemented with 760 equivalent gates or 184 LUTs. Bogdanov et al. [37] proposed a lightweight hash function, named SPONGENT, whose smallest implement cost is 738 equivalent gates. For modular squaring, an estimated implementation cost of 1000 equivalent gates is given in Section 3.4 of Burmester et al. [38]. Table 7 summarizes the hardware implementation costs of these security primitives. Then, we can roughly estimate the implementation cost of each scheme according to the costs of the security primitives. The estimated results are presented in Table 8. From Table 8, we can see that our scheme has the lowest hardware implementation cost and is feasible to be applied in an RFID-based healthcare system with low-cost tags.

### 6.3. Our Proposed Scheme vs. Fan et al.’s Scheme

Based on Section 6.1 and Section 6.2, we highlight the advantages of our proposed scheme by comparing it with Fan et al.’s scheme [14], as summarized in Table 9.

As shown in Table 9, Fan et al.’s scheme [14] cannot meet all the security demands. This scheme fails to assure forward secrecy and cannot resist impersonation attacks, which makes it doubtful to be applied in the real world healthcare systems. Our scheme, on the contrary, can satisfy all the security requirements. When considering the efficiency performance, it is obvious that Fan et al.’s scheme has a much higher overhead than our scheme in terms of computation, communication, and storage costs. For the implementation cost imposed on the tag, our scheme just needs to implement a PRNG while Fan et al.’s scheme needs a PRNG and an additional rotation function. In summary, as an improvement of Fan et al.’s scheme, our scheme demonstrates the superiority in all aspects.

## 7. Conclusions

The legacy healthcare systems have integrated with RFID technology so as to offer better healthcare services. However, the security and privacy concerns about RFID-based healthcare systems are a challenge to combat. In this article, we have analyzed the security of Fan et al.’s scheme [14], a lightweight authentication scheme to secure RFID-based healthcare systems. We first have shown that their scheme is destitute of forward secrecy and also insecure against impersonation attacks. Subsequently, we have proposed an enhanced scheme. Then, we have analyzed the security of the proposed scheme. Analyses illustrate that the proposed scheme can not only overcome the security vulnerabilities of Fan et al.’s scheme but also meet all the essential security demands. In addition, our scheme has low overhead and is compatible with the EPC C1G2 standard. Therefore, our proposed scheme is of practical use for RFID-based healthcare systems.

## Figures and Tables

**Table 1:** Notations.

Notation	Description
p, q	Two large primes
n	n = pq
SID, SID_old_, SID_new_	The tag’s current, previous and next pseudo identifier, respectively
SRID, SRID_old_, SRID_new_	The reader’s current, previous and next pseudo identifier, respectively
x, x_old_, x_new_	The tag’s current, previous and next secret key, respectively
x’	x^2^ mod n, n = pq
y, y_old_, y_new_	The reader’s current, previous and next secret key, respectively
y’	y^2^ mod n, n = pq
T_E_	The current time of E
T_th_	The time threshold
N_E_	The random number generated by E
⨁	The bitwise exclusive-OR
PRNG()	The pseudo random number generator
Rot(x, y)	Left shift x⨁y by y mod L bits, in which L is the length of y

**Table 2:** BAN-logic notations.

Notation	Description
P|≡X	P believes X
P⊲X	P receives X
P|~X	P sends X
P⇒X	P has jurisdiction over X
#(X)	X is fresh
${\left\{X\right\}}_{k}$	X is encrypted by the key k
$P\overset{k}{↔}Q$	P and Q use the shared key k to communicate
$\frac{P}{Q}$	If P then Q

**Table 3:** Security performance comparison.

Scheme	D1	D2	D3	D4	D5
Fan et al. [14]	Yes	No	No	Yes	Yes
Safkhani and Vasilakos [27]	Yes	No	Yes	Yes	No
LRMI [10]	No	Yes	No	Yes	Yes
SecLAP [28]	No	Yes	Yes	No	Yes
Zhou et al. [31]	Yes	Yes	Yes	No	Yes
Our scheme	Yes	Yes	Yes	Yes	Yes

D1: Untraceability; D2: Forward secrecy; D3: Resilience to impersonation attacks; D4: Resistance to desynchronization attacks; D5: Scalability.

**Table 4:** Computation cost comparison (in operations).

Scheme	Tag	Reader	Server	Total
Fan et al. [14]	2 Rot + 4 P	5 Rot + 6 P + 3 MS + SR	2 Rot + 2 Rot^−1^ + 5 P	9 Rot + 2 Rot^−1^ + 15 P + 3 MS + SR
Safkhani and Vasilakos [27]	P + 2 H	P + 2 H	P + 4 H	3 P + 8 H
LRMI [10]	P + 4 C	P + 4 C	P + 4 C	3 P + 12 C
SecLAP [28]	P + 7 MR	P + 17 MR	P + 5 MR	3 P + 29 MR
Zhou et al. [31]	P + H + 3 MS	P + 5 H + 3 MS	6 H + 6 SR	11 H + 6 MS + 6 SR + 2 P
Our scheme	5 P	5 P	9 P	19 P

Rot: rotation operation; Rot^−1^: the inverse operation of Rot; P: pseudo random number generation; H: hash operation; C: cross operation; M: modular rotation operation; MS: modular squaring operation; SR: squaring root solving operation.

**Table 5:** Computation cost comparison (in milliseconds).

Scheme	Tag	Reader	Server	Total
Fan et al. [14]	0.084	9.547	0.105	9.736
Safkhani and Vasilakos [27]	0.527	0.527	1.033	2.087
LRMI [10]	0.021	0.021	0.021	0.063
SecLAP [28]	0.021	0.021	0.021	0.063
Zhou et al. [31]	6.215	6.974	22.404	35.593
Our scheme	0.105	0.105	0.189	0.399

**Table 6:** Performance comparison based on the communication and storage cost.

Scheme	Communication Cost (bits)	Storage Cost (bits)
Fan et al. [14]	2752	1120
Safkhani and Vasilakos [27]	1344	96
LRMI [10]	1632	192
SecLAP [28]	2112	192
Zhou et al. [31]	11008	1120
Our scheme	1344	192

**Table 7:** The hardware implementation cost of the security primitives.

Security Primitive	Implementation Cost (LUTs/Gates)
Rotation function	112/- [14]
Cross function	1/- [10]
Modular rotation function	65/- [28]
Warbler PRNG	184/760 [36]
SPONGENT hash function	-/738 [37]
Modular squaring function	-/1000 [38]

**Table 8:** Performance comparison based on the estimated hardware implementation cost.

Scheme	Security Primitives Used	Implementation Cost (Estimated)
Fan et al. [14]	Rotation function, Warbler PRNG	112 LUTs + 760 Gates
Safkhani and Vasilakos [27]	Warbler PRNG, SPONGENT hash function	1498 Gates
LRMI [10]	Cross function, Warbler PRNG	1 LUT + 760 Gates
SecLAP [28]	Modular rotation function, Warbler PRNG	65 LUTs + 760 Gates
Zhou et al. [31]	Warbler PRNG, SPONGENT hash function, Modular squaring function	2498 Gates
Our scheme	Warbler PRNG	760 Gates

**Table 9:** Performance comparison between our proposed scheme and Fan et al.’s scheme.

	Scheme	Fan et al. [14]	Our Scheme
Performance			
**Security Demands**		Not all satisfied	All satisfied
**Computation Cost**		9.736 milliseconds	0.399 milliseconds
**Communication Cost**		2752 bits	1344 bits
**Storage Cost**		1120 bits	192 bits
**Implementation Cost**		112 LUTs + 760 Gates	760 Gates
